# Swelling Behaviour of Polystyrene Microsphere Enhanced PEG-Based Hydrogels in Seawater and Evolution Mechanism of Their Three-Dimensional Network Microstructure

**DOI:** 10.3390/ma15144959

**Published:** 2022-07-16

**Authors:** Chen Zhang, Yuhong Qi, Zhanping Zhang

**Affiliations:** Department of Materials Science and Engineering, Dalian Maritime University, Dalian 116026, China; pudding@dlmu.edu.cn (C.Z.); zzp@dlmu.edu.cn (Z.Z.)

**Keywords:** swelling behavior, swelling mechanism, three-dimensional network structure, PS-PEG hydrogels

## Abstract

To understand the microstructure evolution of hydrogels swollen in seawater, freeze-drying technology was used to fix and preserve the swollen three-dimensional microstructure. By this method, we revealed the swelling behavior of hydrogels in seawater, and elucidated the mechanism of the swelling process. Meanwhile, we also used Fourier-transform infrared spectroscopy; laser confocal microscopy; field emission scanning electron microscopy, and swelling performance tests to research the structure and properties of PS-PEG hydrogels, before and after seawater swelling, and analyzed the structure and properties of PEG-based hydrogels with different contents of polystyrene microspheres. Results showed that PS-PEG hydrogels went through three stages during the swelling process, namely ‘wetting-rapid swelling-swelling equilibrium’. Due to the capillary effect and hydration effect, the surface area would initially grow tiny pores, and enter the interior in a free penetration manner. Finally, it formed a stable structure, and this process varied with different content of polystyrene microspheres. In addition, with the increase of polystyrene microsphere content, the roughness of the hydrogel before swelling would increase, but decrease after swelling. Appropriate acquisition of polystyrene microspheres could enhance the three-dimensional network structure of PEG-based hydrogels, with a lower swelling degree than hydrogels without polystyrene microspheres.

## 1. Introduction

Hydrogel is a kind of water-containing polymer material that is composed of a physically or chemically cross-linked polymer network and water [1]. Hydrogels have both hydrophilic and hydrophobic groups simultaneously. The hydrophilic group combines with water to lock the water molecules inside the polymer network, and the hydrophobic group swells after absorbing water. Therefore, the water in the hydrogel material loses its fluidity. However, it exists on the network in bonded water, bound water, and free water. Thus, the hydrogel can maintain a particular shape in the water. Hydrogels are generally divided into two categories: one of them is ‘stable chemical gels’. The macromolecules in this hydrogel are generally cross-linked through strong and relatively stable chemical bonds [2]. The degree of equilibrium swelling depends on the interaction parameters and degree of crosslinks of polymer-water [3]. The other one is ‘reversible physical gel’. This type of polymer network mainly relies on entanglement with polymer chains, and some secondary interactions, including ions, hydrogen bonds, hydrophobic interactions, etc. Physical interactions between different polymer chains prevent hydrogel dissolution [4]. Yet, this interaction is reversible and can be easily disrupted by changing physical conditions [5].

The hydrogel system is rich in hydrophilic groups (hydroxyl groups, carboxyl groups, amide bonds, ether bonds, charged groups, etc.) [6]. These materials can absorb hundreds or even thousands of times their weight in water, the 3D network is fully stretched after being swollen, and its volume is expanded many times compared with the dry gel [7]. Gibas and Janik [8] reported that the swelling process of hydrogels is a complex process consisting of several steps. First, the polar hydrophilic groups of the hydrogel are hydrophilized, and primarily bound waters are formed at this time; then, the bound water interacts with the exposed hydrophobic groups, and now secondary bound water appears. Both primary and secondary bound water constitutes the total bound water of the hydrogel; finally, driven by the osmotic pressure, a large amount of free water enters the hydrogel system until equilibrium, the free water in the gel system constantly interacts with that outside. The swelling equilibrium of hydrogels is controlled by external temperature, pH, and physicochemical properties [9]. There are great cross-linking points in hydrophilic molecular chains, and the distance between two cross-linking points is called the cross-linking chain length. In addition, there are many slip rings and physical entanglements between the chains, which determine the hydrogel’s swelling and mechanical properties.

Polyethylene glycol (PEG), as a low-molecular weight water-soluble polyether, is formed by the stepwise addition polymerization of ethylene oxide and ethylene glycol or water. PEG has many advantages, such as good biocompatibility, low toxicity, etc. When other molecules are coupled to PEG, the excellent properties of PEG are also transferred to the conjugate [10,11,12]. Poor mechanical properties are a severe defect of PEG hydrogels. However, most methods to improve mechanical properties can decrease the swelling degree [13]. Therefore, when synthesizing hydrogels, it is necessary to consider various factors affecting hydrogels comprehensively. Tan [14] found that the performance of the gel is affected by the structure of the multi-arm PEG. Eight-arm PEG is more stable and denser than four-arm PEG. Metters [15] prepared PEG hydrogels by Michael’s addition reaction. The study found that the efficiency of crosslinking depends on two parts: the functionality of gel preparation precursor and preparation conditions. Teodorescu [16,17] also confirmed this point of view. Through the study of ‘terminated epoxy PEG and aliphatic diamines with different chain lengths to form gels’, it was found that hydrogels with higher equilibrium swelling degrees were obtained at non-stoichiometric ratios of amine groups to epoxy groups, acidic pH, and lower temperatures. Moffat [18] indicated that hydrogels prepared at low concentrations of cross-linking agents became swollen to a greater degree. Kim [19] synthesized a new type of PEG hydrogel. The PEG macromonomer has two end groups: the sulfonic acid and the methacrylate group. Studies have shown that, PEG hydrogels containing hydrophilic groups have higher swelling ratios than those containing hydrophobic groups. Bignotti [20] reported a modified hydrogel, in which networks contained PEG, poly(propylene glycol) (PPG), and C_18_ alkyl segments. All networks showed an amphiphilic behavior, swelling considerably both in organic solvents and aqueous media. Uyanga [21] found that the optimum microstructure was loaded with polyethylene glycol (PEG) and bismuth telluride (Bi_2_Te_3_) and coated on fabric for imparting thermal sensitivity.

Until now, the hydrogel has been studied and explored to some extent [22,23]. The main problems that existed in the hydrogel material were soft water absorption, reduced strength, and poor adhesion to the substrate [24,25]. These shortcomings limited the application of hydrogels, in some fields with less demanding mechanical properties, such as drug delivery carriers [26], water-absorbing materials [19,27], etc. The practical production of hydrogels has been greatly limited, especially in the marine environment with abundant material resources [28,29]. At the same time, as a soft hydrophilic material, hydrogels had similarities and correlations in the formation of organic molecular layers and biological mucous membranes, which could well block the antifouling phenomenon from the root. However, people had to solve the problems of soft water absorption, low mechanical strength, and lack of long-term antifouling properties, when hydrogel was used to a wide variety of organisms and complex environments in the ocean. So, the use of hydrogel for marine antifouling applications was still in the stage of experimental exploration.

In order to solve problems, we recently reported on a polystyrene (PS) microsphere with a core-shell structure and the preparation process of the composite PEG-based hydrogel; it was amphiphilic and had excellent mechanical properties [30]. In this paper, we combined the previous work to analyze further and characterize the swelling behavior of PS-PEG hydrogels, which before and after swelling in seawater, such as chemical structure, surface morphology, etc. At the same time, the swelling mechanism of PS-PEG hydrogel in seawater was elucidated. Meanwhile, the swelling process was explained and demonstrated by the chemical structure and surface morphology before and after swelling. As we all know, the application of hydrogels in the marine environment was only in the laboratory stage. Our research used intuitive morphology evolution to observe not only the process of hydrogel swelling but also the specific details of each stage, which can assist researchers who elucidate hydrogel simulations to solve swelling problems in related fields. Meanwhile, it also lays the foundation for the exploration of hydrogels in the field of marine antifouling.

## 2. Materials and Methods

### 2.1. Materials

The reagents used in this study were PS-PEG hydrogel, self-made. Styrene (St, CP), purchased from Tianjin Damao Chemical Reagent Co., Ltd. (Tianjin, China). Hydroxyethyl methacrylate (HEMA, AR), polyethylene glycol methyl ether methacrylate (PEGMA, AR), azobisisobutamidine hydrochloride (AIBA, AR), isphorone diisocyanate (IPDI, AR), 1, 4-butanediol (BDO, AR) and polyethylene glycol (PEG) with an average molecular weight of 2000 were purchased from Aladin Biochemical Technology Co., Ltd. (Shanghai, China). Polyether triol (HSH330, CP) was purchased from Jiangsu Haian Petrochemical Plant (Haian, China). Xylene (AR) was purchased from Tianjin Tianli Chemical Reagent Co., Ltd. (Tianjin, China). Ethyl acetate (AR) and cyclohexanone (AR) were purchased from Tianjin Kemiou Chemical Reagent Co., Ltd. (Tianjin, China). NEM conductive tape was purchased from Aladdin Biochemical Technology Co., Ltd. (Shanghai, China); liquid nitrogen was purchased from Dalian West Gas Co., Ltd. (Dalian, China); Silica gel self-indicator (CP) was purchased from Tianjin Hengxing Chemical Reagent Co., Ltd. (Tianjin, China); Seawater was taken from the Yellow Sea (Dalian, China).

### 2.2. Polymerization Process

At first, polystyrene microspheres were prepared by soap-free emulsion polymerization using 20 g St, 250 g deionized water, 4 g HEMA, and 6 g PEGMA as reaction monomers and 0.5 g AIBA as an initiator. Then, 44 g PEG2000 and 10 g IPDI were used as the reactive monomers to conduct the initial reaction in the mixed solvent. Moreover, the 10 g PS microspheres powder prepared in the previous step were added to the system. The -NCO will react with the -OH on the surface of PS microspheres to obtain PS-NCO intermediates through in-situ polymerization. Finally, we used 40 g PEG2000, 10 g IPDI, and 10 g HSH330 as monomers, the isocyanate of IPDI was firstly reacted with the hydroxyl terminations of PEG2000 and HSH330 by solution polymerization method. Then 10 g PS-NCO and 5 g BDO were added to the reaction system, and finally, the PS-PEG polymer solution was obtained. Please refer to the literature [30] for the specific chemical reaction principles and technological processes in detail. 

### 2.3. Preparation of PS-PEG Hydrogel Samples by Moisture Curing 

**Figure 1:**
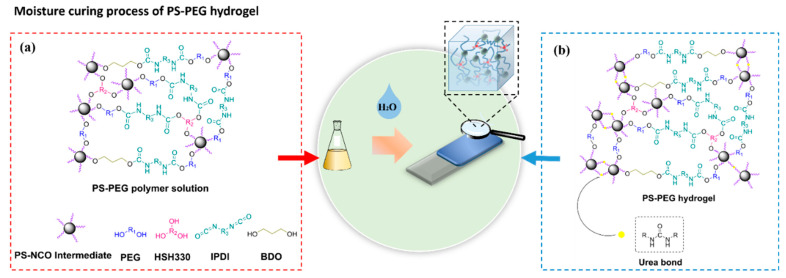
Moisture-curing mechanism of hydrogels: (**a**) isocyanate-terminated PS-PEG polymer solution; (**b**) isocyanate groups react with water to form urea bonds.

The PS-PEG polymer solution was poured into a glass slide with a size of 75 mm × 25 mm × 1 mm and a 200 mm × 200 mm × 10 mm Teflon mold, and it was cured in a dust-free room temperature environment of 72% RH for 2 days. Finally, the PS-PEG hydrogel was obtained after the moisture curing reaction. As shown in Figure 1.

The PS-PEG hydrogel samples were taken out, transferred to a drying dish filled with silica gel self-indicator, and dried for 7 days to ensure that the whole water in the hydrogel was removed, so that the PS-PEG xerogels without seawater swelling (XH) samples were obtained. The glass slide sample was used for laser confocal measurement, and the Teflon mold sample was used for FTIR measurement, seawater swelling test, and scanning electron microscope test.

The PS0-PEG sample was labeled with the ratio of PS microsphere content to monomer mass 0 wt.%. And 7.1 wt.%, 14.2 wt.%, 21.3 wt.%, 28.4 wt.% were labeled PS7-PEG, PS14-PEG, PS21-PEG, and PS28-PEG, respectively.

**Figure 2:**
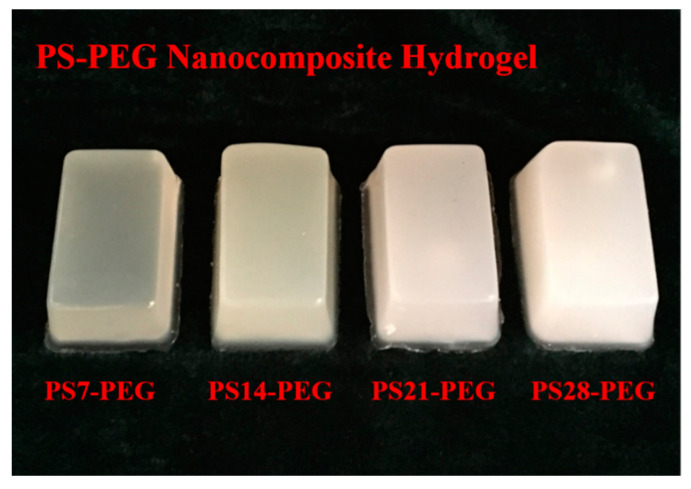
Macroscopic morphology of PS-PEG hydrogels.

The macroscopic morphology of the PS-PEG hydrogel was obtained after moisture curing, as shown in Figure 2. With the increase of the content of PS microspheres, the light transmittance of the hydrogel gradually decreased and changed from translucent to white. 

### 2.4. Swelling Test and Sample Preparation by Freeze-Drying

Seawater was filtered through a SCI-DP12 filter (Shengze Technology Co., Ltd., Tianjin, China) with a 0.22 μm pore size of microporous membrane (φ = 50 mm). Then, it was sterilized with an YX-280-B autoclave (Huatai Medical Equipment Co., Ltd., Hefei, China) for 35 min, cooled to room temperature and taken out for use. The polycarbonate transparent plastic container with a size of 75 mm × 52 mm × 85 mm and a capacity of 200 mL was used, and the sterilized seawater was injected into 100 mL for the hydrogel swelling experiments. The PS-PEG hydrogels obtained 7 days after curing were cut into 2 mm × 2 mm × 1 mm rectangular parallelepiped samples, and swollen in seawater for 48 h. The seawater-swollen hydrogel (SSH) samples were taken out at different time intervals, the water on the sample surface was quickly wiped off with a filter paper, and the samples were placed in a JJ-500 precision balance (Shuangjie Test Instrument Factory, Changshu, China) (accuracy 0.0001) and weighed. The hydrogel swelling degree SD = (WS − W0)/W0. WS and W0 are the masses at which the hydrogel expands over time and back to the original hydrogel, respectively.

In order to reveal the change law in the 3D structure of hydrogels before and after swelling, the state was presented to elucidate the swelling properties intuitively. However, the current observational conditions have not explained the swelling law by the dynamic morphology; problems were often explained through molecular simulations and hypothetical models. Thus, we used the observation of the morphology of PS-PEG hydrogel; after a specific time and the analysis of swelling behavior, the sample was processed by freeze-drying. Then, it was quenched by the liquid nitrogen deep-freezing method. The samples after different treatments were observed, respectively. We have innovatively passed this method and process, although the hydrogel volume may increase due to the expansion of ice during the freezing process. The internal structure can be preserved to the greatest extent, so that the swelling law can be scientifically explained.

**Figure 3:**
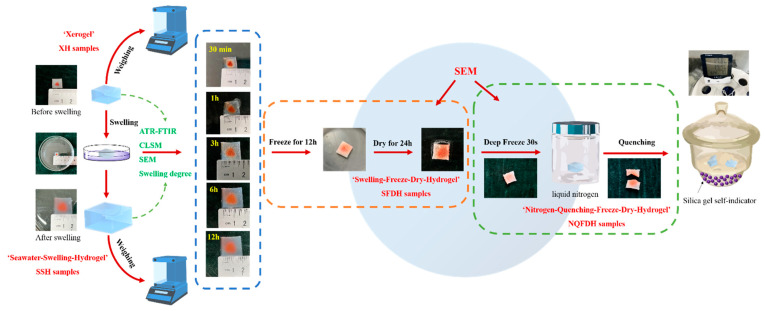
Swelling and freeze-drying preparation process.

The sample preparation process for freeze-drying was shown in Figure 3. The XH samples were cut into 10 mm × 10 mm × 1 mm slices, and swollen in seawater for 30 min, 1 h, 3 h, 6 h, and 12 h. LC-10A-50N vacuum freeze dryer (Lichen Bangxi Instrument Technology Co., Ltd., Shanghai, China) was used to treat SSH samples. Therefore, we obtained the samples which swollen and freeze-dried hydrogels (SFDH). They were taken out and placed in liquid nitrogen for 30 s, then made brittle by natural force, the liquid nitrogen frozen and quenched of the SFDH samples (NQFDH) were obtained. The XH, SFDH, and NQFDH samples were bonded to a metal block (10 mm × 10 mm × 10 mm) with conductive adhesive, and deposited gold within a vacuum chamber for 1 min using a JFC-1100 ION Sputter (Japan Electronics Ltd., Tokyo, Japan).

### 2.5. Fourier Transform Infrared Spectrometer (FTIR)

Taken SSH samples out after swollen 48 h, and the surface was quickly rinsed with deionized water 3 times; it was subsequently dried with a hairdryer for 2 min to remove interference, such as seawater ions and water. The XH and SSH samples were tested by FTIR (PerkinElmer, Waltham, MA, USA). The hydrogels’ chemical structure was analyzed in the attenuated total reflection mode, with the scanning range of 4000 to 650 cm^−1^, the resolution of 2 cm^−1^, and the number of scans was 32 times.

### 2.6. Morphology

#### 2.6.1. Scanning Electron Microscope (SEM)

The morphology and characterization of XH, SFDH, and NQFNH samples were observed with a Supra-55-sapphire SEM (Carl Zeiss AG, Jena, Germany). Meanwhile, the current mode was set to DC, the voltage was 5 kV, the current was 5 mA, the observation mode was SE2, and the accelerating voltage was 1 kV.

We used ‘Image pro plus’ software to measure hydrogel samples ‘mean pore diameter’ (Dm). It means, average length of all line segments intersecting the object measured at 2 degrees intervals through the object’s centroid. The ‘manual mode’ was used for the measurement method; the ‘gray scale 16′ and the ‘best fit’ mode were used for preprocessing.

#### 2.6.2. Confocal Laser Scanning Microscope (CLSM)

The surface and fracture of the XH and SSH samples were observed and analyzed by OLS4000 CLSM (Olympus, Tokyo, Japan). LEXT analysis software was used to measure the line roughness (Ra) and surface roughness (Sa).

## 3. Results

### 3.1. Surface Morphology of the Hydrogels

**Figure 4:**
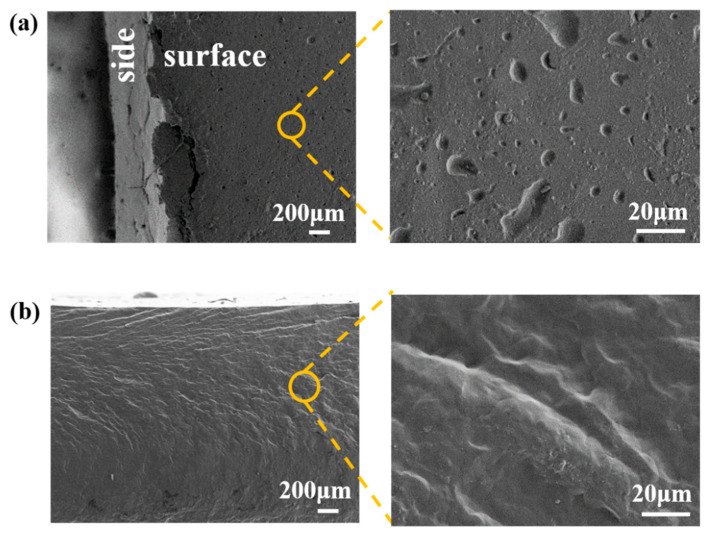
SEM morphology of PS14-PEG XH sample: (**a**) surface; (**b**) fracture.

For XH samples, neither the surface nor the fracture morphology differed. So, we selected PS14-PEG hydrogel as the representative sample. The surface of hydrogel was dense morphology with many pits, as shown in Figure 4a, because the PS-PEG hydrogels were isocyanate-terminated polymer solutions before moisture curing; during this process, water molecules acted as chain extenders, consuming a large amount of isocyanate to form urea bonds, at the same time, producing a large amount of carbon dioxide, which would create irregular pits on the surface where these gases escaped.

The fracture was smoother and more compact than the surface, as shown in Figure 4b, and there was clear texture. At this time, PS microspheres were used as cross-linking centers inside the hydrogel, and the cross-linking points of PEG-based hydrogels were generated after in-situ polymerization. Due to the uniform distribution of PS microspheres and macromolecular chains in the system, the densely cross-linked structure was formed in the relatively regular microstructure. These networks made up of polymers ensured that the solvent would not dissolve the hydrogel.

### 3.2. Swelling Behavior and Mechanism in Seawater

#### 3.2.1. Swelling Behavior

**Figure 5:**
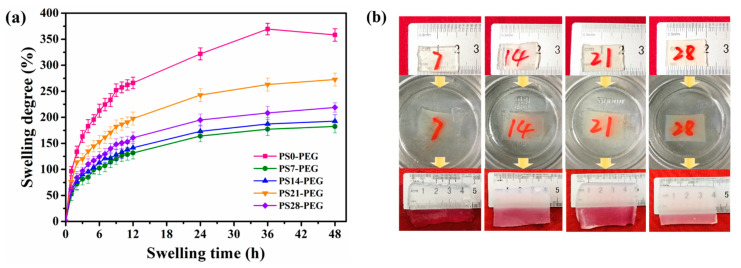
Swelling behavior of hydrogels in seawater: (**a**) Swelling degree vs. swelling time; (**b**) Specimen photos in the experimental procedure.

The swelling properties of PS-PEG hydrogel placed in seawater for 48 h were shown in Figure 5. The results showed that the hydrogel without PS microspheres had the highest swelling degree in seawater, reaching 358.67% after 48 h, while the PS7-PEG sample had the lowest in the test, only just 182.5%. After the third polymerization step, a denser 3D cross-linked structure was formed than hydrogel without microspheres. However, the limited space would hinder the absorption of seawater by the hydrogel. The excess seawater was squeezed out and gradually reached saturation. Therefore, PS-PEG hydrogels were generally less swollen than those without microspheres. At the same time, the swelling degree was not increased with the addition of PS microspheres. 

On the contrary, it was observed that the swelling degree of PS28-PEG hydrogel was lower than PS21-PEG hydrogel. This was because too many PS microspheres made the internal cross-linked structure higher, which was sterically confined to a greater extent, so the swelling degree decreased instead. In the initial 3 h of swelling, the swelling degree of each sample increased rapidly, and the PS0-PEG hydrogel had the highest swelling property. The sample contained numerous hydrophilic groups such as -OH and -CONH-. Thus, seawater reacts with these ester groups, carbamate groups, and urea groups, resulting in PS0-PEG hydrogel having a higher swelling degree than other PS microsphere-added hydrogels. Interestingly, the hydrogels’ swelling degrees in deionized water for 48 h respectively reached 239.78%, 246.90%, 339.64% and 284.62%. The swelling degree of the samples with PS microspheres added in seawater was lower than that in deionized water [30]. There were two reasons: on the one hand, the sodium ions and chloride ions in seawater penetrated the hydrogel system, which reduced the binding ability of the hydrogel to seawater; on the other hand, the high osmotic pressure generated by the high ionic strength of seawater environment was applied to the hydrogel system, thereby reducing the swelling equilibrium of the hydrogel. Meanwhile, as can be seen from Figure 5a, the hydrogels added with PS microspheres maintained stable swelling after swelling equilibrium.

SSH samples were swollen in seawater for 48 h, and the morphological comparison before and after were shown in Figure 5b. Moreover clearly, hydrogels swollen to a particular volume in seawater, and the macroscopic was also changed.

#### 3.2.2. Chemical Structure Evolution during Swelling 

**Figure 6:**
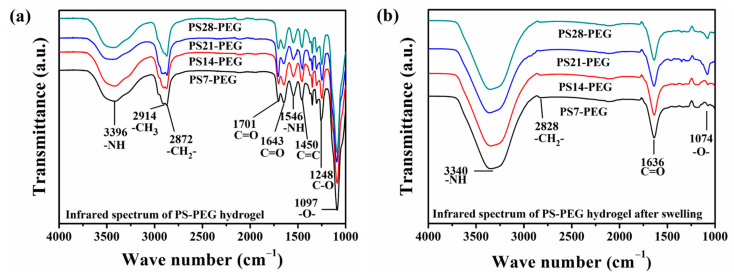
Infrared spectra of the hydrogels: (**a**) XH; (**b**) SSH.

ATR-FTIR spectra XH and SSH samples were shown in Figure 6. It can be observed that some of the characteristic peaks after swollen in seawater had an apparent red-shift phenomenon. The stretching vibration absorption peak of -NH was at 3396 cm^−1^ in Figure 6a, but the red-shifted to 3340 cm^−1^ after swelling, caused by the hydrogen bonding of free -NH with carbamate. When the hydrogel was swollen, the hydrophobic groups on the surface would move to the inside with the inversion of the flexible chain, such as the methyl characteristic peak at 2914 cm^−1^, etc. Only the weak absorption peak of the methylene group at 2828 cm^−1^ could be measured in Figure 6b. At the same time, the 1643 cm^−1^ and 1701 cm^−1^ shown in Figure 6b was the stretching vibration absorption peaks of C=O in the urea group and the amide bond. After swollen in seawater, the carbonyl peak was also red-shifted to 1638 cm^−1^, and no longer split into two peaks. Some absorption peaks had weak intensities and the flipping of the flexible chain, which would cause them to have no significant characteristics after swollen in seawater, such as the amino deformation vibration peak at 1546 cm^−1^ and the carbonyl absorption peak at 1450 cm^−1^, etc. Meanwhile, the ether bond also had a red-shift after swollen in seawater, from 1097 cm^−1^ to 1074 cm^−1^.

In summary, after 48 h of swelling in seawater, the characteristic absorption peaks of the amino bond, carbonyl bond, and an ether bond of the hydrogel all red-shifted. The main reason was that water molecules in seawater could provide -H acceptors, thereby increasing the degree of hydrogen bonding. The formation of hydrogen bonds increased the polarization of the chemical bond, which changed the force constant of the two groups. Therefore, the frequencies of both stretching and bending vibrations were altered accordingly. However, as the content of PS microspheres increased, there was no apparent difference in the absorption peaks of the hydrogels, because only the strength of the cross-linked hydrogel structure was improved.

The above analysis showed that, for SSH samples, -NH and -CO- tended to form hydrogen bonds with water molecules, which increased the number of hydrogen bonds. However, the number of hydrogen bonds on the segmental structure was reduced. Thereby, the degree of hydrogen bonding with the hydrogel structure chains would be weakened.

#### 3.2.3. Surface Morphology Evolution during Swelling

**Figure 7:**
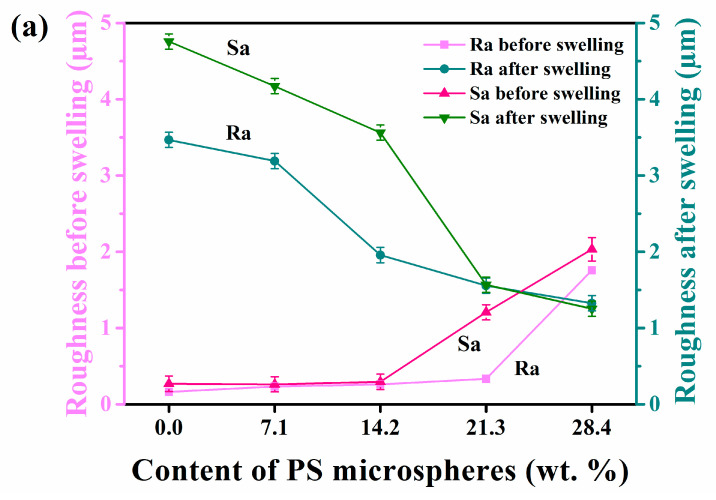

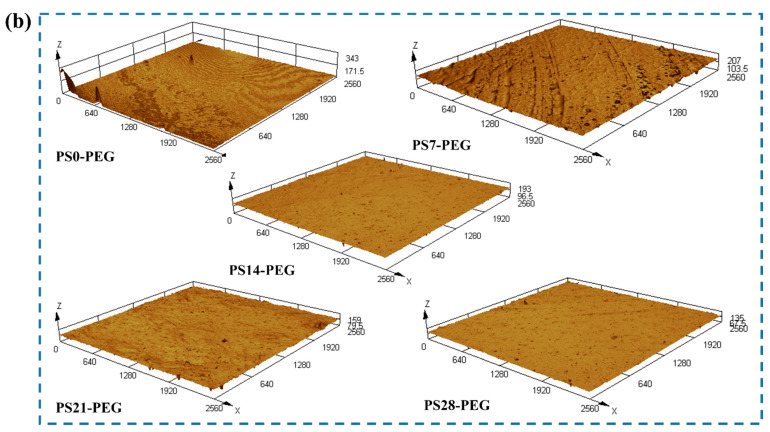
Surface roughness and 3D morphology of the samples: (**a**) Roughness of XH and SSH samples, (**b**) 3D surface morphology of the SSH samples.

A trend line was drawn based on the line (surface) roughness measurement of XH and SSH samples, as shown in Figure 7a. The Ra and Sa of PS0-PEG hydrogels were the lowest for the XH samples, while other samples increased with PS microspheres. The reason was that the particle size of PS microspheres was small, which caused high surface activity. Too many nano-microspheres would promote the agglomeration effect. It was easy for agglomerations to form in some areas not wholly dispersed, resulting in increased roughness. From the observation and analysis of morphology, the unevenness of its surface was irregular.

Interestingly, when SSH samples were swollen for 48 h, the roughness change trend was reversed. The Ra and Sa of each sample decreased by increasing of PS microspheres. Because PS microspheres were the cross-linking points in the hydrogel, this resulted in increased crosslink density and tighter 3D space. When the hydrogel was in contact with water from the air interface, under the strong polarity of seawater, the flexible chains of the internal hydrophilic groups could be rapidly reconfigured and flipped. A hydration layer was formed on the surface, thus reducing the surface roughness. PS-PEG hydrogels with different amounts of PS were photographed in macroscopic appearance, as shown in Figure 7b. With the increase of PS microspheres, the surface was smoother after being swollen at the same time.

##### Effect of Swelling Time in Seawater on Swelling Structure

**Figure 8:**
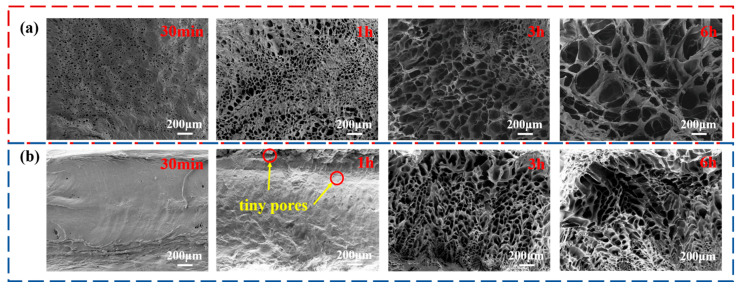
Morphology of PS14-PEG SFDH sample with different swelling time: (**a**) surface, (**b**) fracture.

This research showed that, as the increased seawater swelling time, all hydrogel surfaces were first swollen with water. The seawater continued to penetrate the interior, until it had permeated through the entire sample, and finally reached the swelling equilibrium. Along with this process, we took the PS14-PEG SFDH sample as an example; changes in the law of the 3D structure of surface and fracture are shown in Figure 8.

When the SFDH samples were swollen for 30 min, which was the initial swelling stage, water molecules entered the hydrogel and formed dense pores on the surface. When the SFDH samples were swollen for 1 h, as the hydrogel continued to swell, the tiny pores on the surface overlapped and gradually formed large pores. At this time, the surface of the SFDH samples began to be decomposed and degraded by the pores, and the seawater continued to infiltrate inwards due to osmotic pressure. However, the fracture had not changed obviously due to the different penetration rates. There were only tiny pores in the near-surface area, where as shown the red circles in Figure 8b. When the SFDH samples were swollen for 3 h, they entered the rapid swelling period. During this process, the SFDH samples continued to absorb seawater until the swelling equilibrium. The 3D network structure was continuously improved, new pores continued to grow and expand, and finally formed a rich and dense stable system around 6 h.

**Figure 9:**
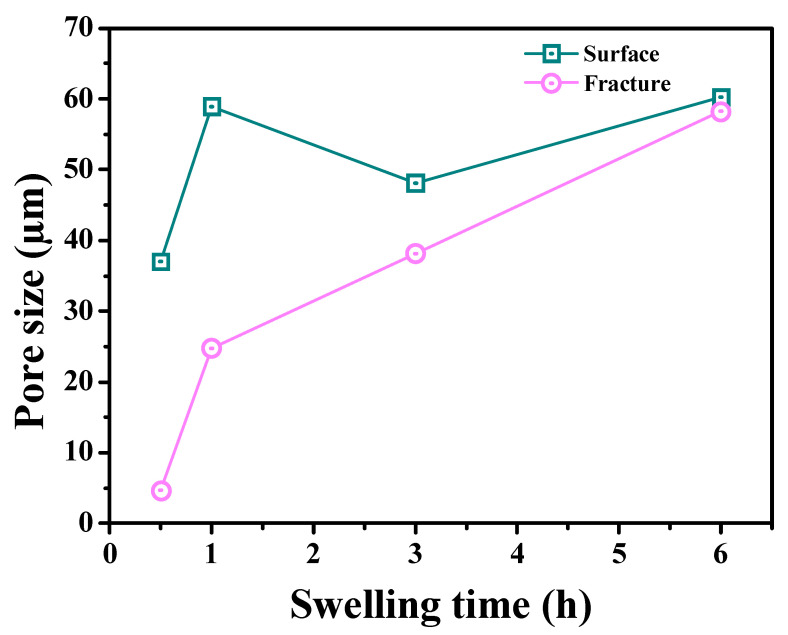
Effects of swelling time on pore size for PS14-PEG hydrogel.

Through the calculation of the Dm from PS14-PEG SFDH surface and fracture at different swelling times, the results were shown in Figure 9. It can be seen that the pore size on the surface and fracture increased with the swelling time, and the process on the surface was more complicated than the fracture. In the growing trend of fracture pore size, the Dm has been increasing. The pore size increased by 20.12 μm when the hydrogel was swelling from 30 min to 1 h, the growth rate was 40.24 μm/h; it increased by 13.36 μm within 1 h to 3 h, and the growth rate was only 6.68 μm/h; meanwhile, the pore size only increased by 20.11 μm within 3 h to 6 h, and the growth rate at this stage was only 6.7 μm/h. Therefore, it can be concluded that the growth rate of the latter two stages was much lower than the first 1 h.

However, the trend of surface pore size growth was different. The Dm increased from 37.07 μm to 58.98 μm within 30 min to 1 h, and the growth rate was 43.82 μm/h. But in the following 1 h to 3 h, the Dm decreased by 10.83 μm, which was inconsistent with the common law. This was due to the continuous tiny pores on the pore walls, with smaller and denser pore size, resulting in a lower Dm. Nevertheless, during the 3 h to 6 h, the Dm of the hydrogel surface increased by 12.12 μm, and the growth rate was only 4.04 μm/h. Compared with the trend in the fracture, the Dm of the surface behaves more slowly in this period, and the rate of pores was lower, which varied from tiny to large.

In summary, the Dm of PS14-PEG hydrogel has been on an upward trend with the increase in swelling time, and the growth rate both on the surface and the fracture has a maximum value of 30 min to 1 h.

##### Effect of PS Microspheres Addition on Swelling Surface Microstructure

**Figure 10:**
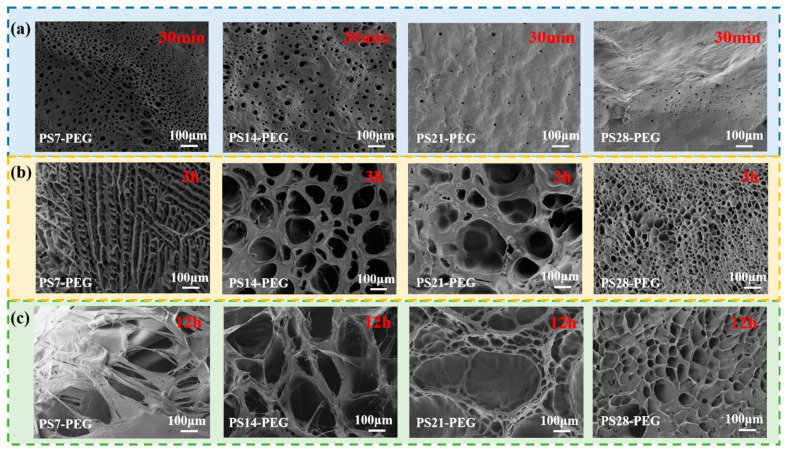
Surface morphology of the SFDH samples: (**a**) 30 min, (**b**) 3 h, (**c**) 12 h.

The surface morphologies of the SFDH samples swollen for 30 min, 3 h, and 12 h are shown in Figure 10. It can be observed from Figure 10a that the surface morphology was different after swelling for 30 min. In the early stage of swelling, with the increase in the content of PS microspheres, the pore density of the surface decreases. This was because the PS microspheres increased the cross-linked structure of the hydrogel, the dense network reduced the capillary effect and the hydration effect, so that the rate of entry of water molecules became slow. When the SFDH samples entered the rapid swelling period, as shown in Figure 10b, numerous water molecules entered due to the action of osmotic pressure, and the network pore size was continuously expanded at this time. However, the SFDH samples with different PS microsphere contents affected the porosity and pore size changes. When the SFDH samples entered the swelling equilibrium period, the cross-linked structure inside the hydrogel can provide elastic contraction force, to resist the swelling caused by the osmotic pressure of the hydrogel itself. On the contrary, since the hydrogel had a poor cross-linked network structure, its pore walls were continuously expanded and weakened, finally forming filaments and no longer maintaining their original strength. The surface morphologies of the SFDH samples swollen for 12 h were shown in Figure 10c. PS microspheres made the network structure of the hydrogel more compacted. The larger the pore size of the hydrogel network structure, the higher the water absorption rate. On the contrary, the smaller the pore size, the lower the water absorption rate. It was apparent in the figure that, the PS21-PEG SFDH sample had the largest pore size and the strongest water absorption rate, which was also consistent with the swelling curve in Figure 5a.

##### Effect of PS Microspheres Addition on Fracture Morphology

**Figure 11:**
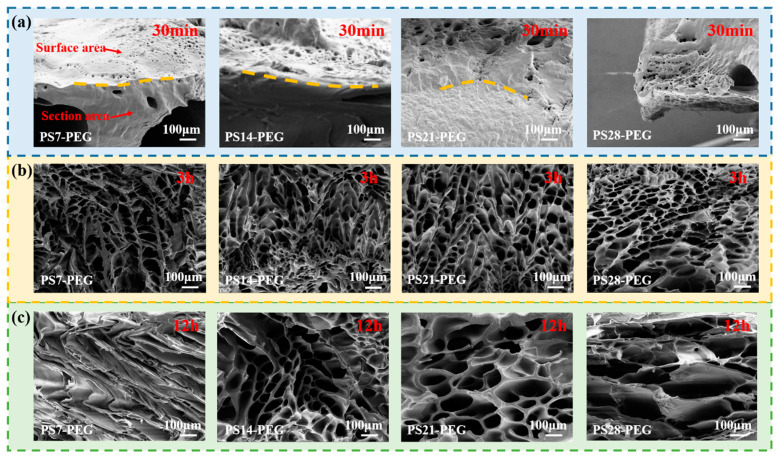
Fracture morphology of the NQFDH samples: (**a**) 30 min, (**b**) 3 h, (**c**) 12 h.

After the NQFDH samples were swollen in seawater for 30 min, 3 h, and 12 h, the fracture morphologies were shown in Figure 11. It was different compared with the XH samples fracture in Figure 4b. It can be found by comparison that at the initial stage of swelling, as shown in Figure 11a, tiny pores appeared on the surface of the hydrogel, due to the existence of the capillary effect and hydration effect. However, the interior of the hydrogel was still in an unswollen state, indicating that the swelling process was penetrating from the surface to the interior. When the NQFDH samples became swollen rapidly, as shown in Figure 11b, numerous water molecules entered the hydrogel due to osmotic pressure. Because of the influence of the content of PS microspheres, when fractured by an external force, the NQFDH samples had different degrees of cross-linking structure; thus, they became deformed differently with the direction of the external force. When the NQFDH samples were swollen by 12 h, the fracture of the hydrogel with different PS microsphere contents were obtained again. As shown in Figure 11c, the cross-linked network structure of the hydrogel had been formed. The network of weak structural strength would not be able to maintain the original structural shape due to the force, resulting in large deformation. Especially PS7-PEG NQFDH sample showed a branch-like morphology. PS microspheres not only had a rigid structure, but also acted as cross-linking points of the hydrogel network through in-situ polymerization. Therefore, the 3D network structure of the hydrogel could be significantly enhanced, so hydrogels with a higher content of PS microspheres could maintain a better system.

According to previous work [30], PS-PEG hydrogels have excellent mechanical properties. The appropriate content of PS microspheres can improve the mechanical properties of PS-PEG hydrogels. When the content of PS microspheres is at 14.2 wt.%, the maximum compressive stress reaches 5.83 MPa, the relative compression ratio reaches 161.96%, and the compressive elastic modulus is 73.7 kPa. Therefore, under the external force, PS-PEG hydrogel could maintain the integrity of the internal structure.

Combined with the different XH, SSH, SFDH, and NQFDH sample morphologies during the swelling process, it can be divided into three stages, ‘initial swelling stage’, ‘rapid swelling stage’ and ‘swelling equilibrium stage’. The main feature of the initial swelling (0–30 min) was the appearance of small and dense porous structures on the surface, the water in the environment was combined with the hydrogel at this time, and the hydrophilic group inside hydrogel was flipped through, and the flexible chain tended to surface. Hydrogel surface entered the stage of wetting. Meanwhile, the capillary effect and hydration effect were dominant. Water molecules gradually formed tiny pores in the surface layer, and the volume began to expand. When the hydrogel entered the rapid swelling period (30 min–6 h), at this stage, the hydrogel would absorb water rapidly, and the influence of osmotic pressure was dominant. So, it caused the volume to expand, and the elastic contraction force to gradually increase, while the pores would be denser, and pore size would also increase. Hydrogel would go through two processes successively; the first process was ‘surface layer degeneration–pore size growth–formation of preliminary network structure’ (0.5–1 h), water molecules continued to enter the hydrogel, and the denser pores led to continuous fading of the surface layer. As the pore size continued to grow, pore walls were formed there, and then formed a preliminary network structure. The second process was the enrichment of the network structure, and new pores continued to grow on the pore walls (1–6 h). This process was further divided into two cases. When the cross-linked structure was low, the elastic shrinkage force was weak at this time, and the hydrogel tended to swell infinitely. This resulted in the gradual thinning of the pore walls, which eventually formed a filamentous shape, which collapsed during the freeze-drying process, because it could not support the structure. When the cross-linked structure was high, the elastic contraction force was strong at this time, and the hydrogel tended to swell to equilibrium. The pore wall was eventually stabilized, and the structure was compact, which could maintain the stability of the internal structure during the freeze-drying process. When hydrogel entered the swelling equilibrium period (≥12 h), at this stage, the osmotic pressure and elastic contraction force were in a dynamic equilibrium state, hydrogel structure was basically stable, and the swelling degree remained constant. If the cross-linked network structure was weak, hydrogel tended to swell infinitely, causing the surface to be damaged by hydration. The internal structure was easily pulled to break by an external force, and finally showed a tree branch shape. If the cross-linked network structure was strong, the hydrogel surface structure tended to be stable, ensuring that the inner structure was not damaged when the internal structure was subjected to an external force.

**Figure 12:**
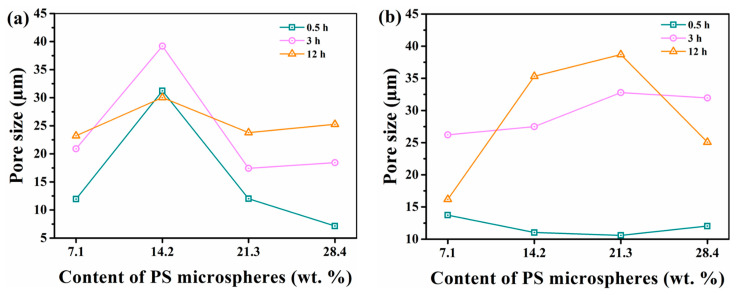
Effects of PS microspheres on pore size at different time: (**a**) Surface; (**b**) Fracture.

With the increase of the content of PS microspheres, the Dm of both the surface and fracture changed significantly. In Figure 12a, the Dm on the surface did not always increase with the PS microspheres. In contrast, for the same swelling time, the Dm of PS21-PEG and PS28-PEG samples were even lower than those of PS14-PEG. It was Dm (PS14-PEG) > Dm (PS28-PEG) > Dm (PS21-PEG). This was due to the role of PS microspheres as cross-linking points in in-situ polymerization. When the content of PS microspheres added increased, new pores continued to grow on the pore walls during the swelling period, thus reducing the Dm to a certain extent. However, when swelling for 12 h, the Dm of each sample at swelling equilibrium was small. It was worth noting that the Dm of PS14-PEG was lower than 3 h. It could be observed from Figure 10 that with the increase in swelling time, the filamentation of its pore walls appeared and gradually increased. The hydrogel could not maintain the original pore-like network structure, so the Dm decreased. 

The Dm of the fracture with different PS microsphere additions changed considerably. In the early stage of swelling (within the first 30 min), the water absorption process first existed on the surface, with low impact on its interior. Therefore, there was little difference in Dm between the samples after 30 min. However, when swollen for 3 h, different from the trend presented on the surface, the Dm rose with the increased PS microsphere content. At this time, Dm (PS21-PEG) > Dm (PS28-PEG) > Dm (PS14-PEG). Interestingly, the Dm of each hydrogel again showed a significant difference when swelling for 12 h. The Dm of PS7-PEG hydrogel decreased by 10.02 μm. That was, as the swelling of the hydrogel tends to equilibrium, the law by which the pore size maintains a certain pore size did not reflect. Combined with the corresponding SEM in Figure 11, it can be concluded that due to the excessive swelling of the PS7-PEG hydrogel, the original pore structure could not be maintained under external stress, and a few PS microspheres can make the 3D network crosslinking structure of the hydrogel more compact. However, the number of connection points was limited and the crosslinking structure was not perfect, so the segment with weak bonding strength displayed breaks more often. Meanwhile, the hydrogel will deform under the action of natural forces, in the place where the spatial structure of hydrogel was not closely cross-linked, the low relative bond strength place made the limit easier to reach, resulting in a break. The fracture occurred in the position where the relative strength of the hydrogel bonding was poor, so the Dm was also decreased. At the same time, the Dm of PS28-PEG also decreased by 6.88 μm compared with 3 h. This was due to the excess of PS microspheres, resulting in a longer swelling process and a correspondingly more extended transition period at the same swelling time.

In conclusion, for hydrogels with different content of PS microspheres, the surface and the fracture showed two opposite swelling effects during the same swelling time. The addition of PS microspheres can lead to the increase in cross-linking sites in the hydrogel, thereby affecting the structural strength of the hydrogel, and the difference was pronounced when subjected to an external force [30].

#### 3.2.4. Swelling Mechanism

**Figure 13:**
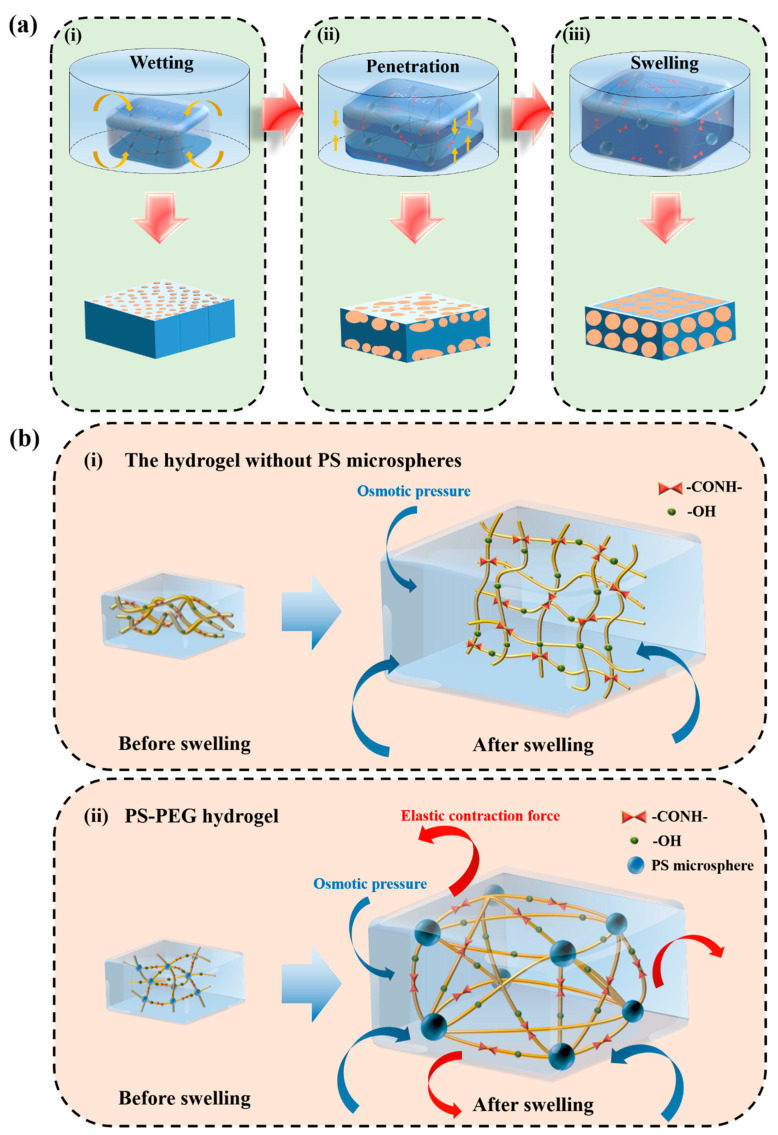
The swelling mechanism of the hydrogel: (**a**) three stages model of the XH samples swelling in seawater at different time: (i) wetting; (ii) infiltration; (iii) swelling equilibrium, (**b**) effect of PS microspheres: (i) PEG hydrogel without PS microspheres; (ii) PS-PEG hydrogel.

When PS-PEG XH samples were placed in seawater from a dry state, there would experience ‘wetting’ processes first. If the surface was in contact with water, numerous hydrophilic groups would form a glass-like tube, such as hydroxyl groups. The capillary effect was generated, which caused the water to diffuse into the gap continuously [30,31,32]. Due to the combined effect of hydrogen bonding and hydration, the hydrogel was further wetted. As shown in Figure 13a(i), the hydrophilic group on the hydrogel surface would capture water molecules first, and this part of the water was called ‘bound water’. It was firmly bound to numerous hydrophilic groups in the form of hydrogen bonds [33]. Many dense pores appeared on the surface during the wetting process, and the specific surface area increased. However, the water absorbed by capillary and the hydration effects were orders of magnitude worse than that absorbed by hydrogels. After all, osmotic pressure and network structure were the most important reasons for the formation of highly hydrated hydrogels [34]. When the PS-PEG hydrogel absorbed water, it entered the ‘penetrated’ process, as shown in Figure 13a(ii). The polar hydrophilic groups in the hydrogel were hydrophilized, and combined with water molecules to form ‘primary bound water’. Then, these bound waters interacted with the hydrophobic groups in the groups, and at this time ‘secondary bound water’ appeared. They constitute the total bound water of the hydrogel. In this process, the bound water obtained by interaction was not combined, but existed in the form of a weak force such as van der Waals force [35]. In the process of penetration, the swelling always maintained the process from surface to inner, at this time, the pores of hydrogel began to grow continuously and develop towards a network trend. PS-PEG hydrogels entered the swelling equilibrium after a while, and the hydrogel structure was stable. As shown in Figure 13a(iii), it did not dissolve with swelling. As the internal hydrogel structure continued to swell during this stage, the pressure inside the hydrogel decreased. Due to osmotic pressure, the network would absorb additional water, which was called ‘free water’ [36]. This extra water absorbed by swelling was in stark contrast to bound water, via chemical or physical bonds. This part of the water molecule was only distributed in the network chains. The free diffusion was not affected by the polymer, yet played a significant role in the swelling volume of the hydrogel. In addition, the hydrogel was also made more elastic due to the entry of additional water [26].

In general, before the XH samples absorbed seawater, the long chains of macromolecules were drawn close, intertwined, and cross-linked to form a network structure. Since the polymer network was a solid structure, it was not ionized into ion pairs. In PS-PEG hydrogels, the absorbed water would hydrate with -CONH- groups and hydration produced an environment different from free water, so due to the increased osmotic pressure difference between the environment inside and outside, the water molecules also penetrated a network structure, thus endowing PS-PEG hydrogels with powerful water absorption capacity. Meanwhile, the seawater contained ions such as Na^+^, K^+^, Cl^−^, and these anions and cations would reduce the binding ability of hydrophilic groups such as -CONH- and -OH to water. The high ionic strength outside the hydrogel system produced high osmotic pressure, and applied to the hydrogel system would reduce the swelling equilibrium of hydrogel [37,38,39]. The lower ion concentration of the external environment, the inside, and outside of the cross-linked network had more significant ion concentration differences, and tremendous osmotic pressure as well. However, when the external environment was deionized water, the osmotic pressure was low due to few ions. In order for concentrations between internal and external ions to be the same, a large amount of water was required to enter the network of the hydrogel, so the water absorption rate was generally high.

However, this absorption and swelling were not infinitely extended, the extent of which depended mainly on the internal cross-linking of the polymer. This swelling process of polymers was an equilibrium process, with the two opposite trends of water absorption and swelling. The solvent tried to penetrate the interior of the network structure, causing the volume to swell and the 3D structure to stretch. The molecular chain at its cross-linking point was stretched, so that its conformational entropy decreased [40]. Meanwhile, there will be another reaction force between the networks, which can cause the molecular network to contract, preventing the hydrogel from increasing in volume, that is, the elastic contraction force. These two forces act at the same time, but in opposite directions. When the magnitudes of their acting forces are the same, they can cancel each other out and achieve swelling equilibrium. The lack of sufficient cross-linking points for hydrogels without PS microspheres made the cross-linking structure sparse, and the swelling effect was substantial. The elastic contraction force was weak at this time, as shown in Figure 13b(i). While PS-PEG hydrogels added PS microspheres, the surface of PS microspheres had numerous reactive groups, and participated in the in-situ polymerization reaction, so that the hydrogel had a densely cross-linked network structure, and PS microspheres acted as cross-linking fulcrums. When PS-PEG hydrogels were swollen in water, the densely cross-linked structure resulted in increased elastic contraction force. That was to say, the degree of shrinking the structure was increased from swelling. Therefore, when the PS-PEG hydrogel reached the swelling equilibrium, its swelling degree was much lower than without PS microspheres, as shown in Figure 13b(ii).

## 4. Conclusions

The structure and properties of PS-PEG hydrogels before and after swelling in seawater were tested and analyzed. Freeze-drying was used to fix and preserve the 3D microstructure of hydrogels swollen in seawater, and to elucidate the mechanism of the swelling process. The results show that seawater could increase the degree of hydrogen bonding with the hydrogel, and then the infrared absorption peak shifted to the low frequency region. Still, the amount of PS microspheres had no effect, but in surface roughness, the XH samples surface roughness increased with the content of PS microspheres, and the surface roughness of SSH samples was reversed. In addition, ions in seawater reduced the water-binding ability of the hydrophilic groups of SSH samples. At the same time, with the high osmotic pressure produced by ionic strength outside the hydrogel system, it was possible to reduce the swelling equilibrium. When PS microspheres were added at 14.2%, PS14-PEG hydrogels had the lowest average swelling degree of 114.45% in seawater. On this basis, the swelling process of hydrogels in seawater was divided into three parts; the first was the ‘wetting’ process caused by the capillary effect and the hydrophilic effect; the second was the ‘rapid swelling’ process caused by the osmotic pressure, and in this stage the swelling always remained a process from the surface to the interior; finally, the process of reaching ‘swelling equilibrium’ when the outward expansion force was the same magnitude as the elastic contraction force. Adding PS microspheres as cross-linking fulcrums can increase the 3D network structure of hydrogel, resulting in a lower swelling degree than that without PS microspheres.

## Data Availability

The data in this study are contained within the article.
